# Does pregnancy increase the risk of ART-induced hepatotoxicity among HIV-positive women?

**DOI:** 10.7448/IAS.17.4.19486

**Published:** 2014-11-02

**Authors:** Susie Huntington, Claire Thorne, Jane Anderson, Marie-Louise Newell, Graham Taylor, Deenan Pillay, Teresa Hill, Pat Tookey, Caroline Sabin

**Affiliations:** 1 Department of Infection & Population Health, UCL, London, UK; 2 Population, Policy and Practice Programme, UCL Institute of Child Health, London, UK; 3 Centre for the Study of Sexual Health and HIV, Homerton University Hospital NHS Foundation Trust, London, UK; 4 Human Development and Health, University of Southampton, Southampton, UK; 5 Faculty of Medicine, Imperial College, London, UK

## Abstract

**Introduction:**

High rates of hepatotoxicity have been observed among HIV-positive pregnant women using antiretroviral therapy (ART). However, the extent to which pregnancy affects the risk of ART-induced hepatotoxicity is unclear since studies in this area have generated conflicting results.

**Material and Methods:**

Combined data from the UK Collaborative HIV Cohort (UK CHIC) study and the UK and Ireland National Study of HIV in Pregnancy and Childhood (NSHPC) were used. Alanine aminotransferase (ALT) data were assessed according to the Division of AIDS toxicity guidelines to identify factors associated with liver enzyme elevation (LEE) (grade 1–4). Women starting ART in 2000–11 aged 16–49 years were included irrespective of pregnancy status at ART start. Cox proportional hazards were used to assess the associations between fixed (ethnicity, exposure group, HBV/HCV co-infection, prior ART use, and age, year, pregnancy status, viral load and CD4 count at ART start) and time-dependent covariates (pregnancy status, age, year, CD4 count, viral load, duration on ART) and the risk of LEE.

**Results:**

Of the 3426 women included, one-quarter (25.0%, *n*=857) were pregnant during follow-up and 14.4% (*n*=492) started ART during pregnancy. The rate of LEE was 15/100 person-years (PY) during pregnancy and 6.1/100 PY outside pregnancy. The risk of LEE was increased during pregnancy (adjusted hazard ratio (aHR) 1.61 [1.26–2.06], *p*<0.001), including in secondary analysis excluding 493 women pregnant when starting ART. Other factors independently associated with LEE were lower CD4 count (<250 cells/mm^3^ vs. 251–350 cells/mm^3^ aHR 1.25 [1.02–1.54], *p*=0.03), HBV/HCV co-infection (aHR 1.94 [1.58–2.39], *p*<0.001), HIV acquired via injecting drug use (aHR 1.61 [1.15–2.24], *p*=0.01 vs. heterosexually) and calendar year (aHR 1.05 [1.02–1.08], *p*<0.001 per one year increase). Three ART drugs were associated with increased risk of LEE (efavirenz aHR 1.27 [1.06–1.50], p-value 0.008; maraviroc 4.19 [1.34–13.1], *p*=0.01; and nevirapine 1.59 [1.30–1.95], p-value <0.001). Use of zidovudine was associated with decreased risk of LEE (aHR 0.74 [0.63–0.87], *p*<0.001) as was increasing time on an NNRTI-based regimen (aHR 0.91 [0.86–0.96], *p*<0.001 per additional year).

**Conclusions:**

Pregnant women were at increased risk of LEE, highlighting the importance of close monitoring of toxicity biomarkers during pregnancy.

